# Editorial: The Role of Microbiota in the Onset and Development of Intestine and Liver Diseases and Cancer: Molecular and Cell Mechanisms

**DOI:** 10.3389/fcell.2022.852188

**Published:** 2022-03-09

**Authors:** Fausto Andreola, Camilla Moliterni, Andrea Quagliariello, Franco Scaldaferri, Marco Fidaleo

**Affiliations:** ^1^ Liver Failure Group, Institute for Liver and Digestive Health, Royal Free Hospital, University College London, London, United Kingdom; ^2^ Department of Biology and Biotechnology Charles Darwin, University of Rome Sapienza, Rome, Italy; ^3^ Department of Comparative Biomedicine and Food Science, University of Padova, Padova, Italy; ^4^ Dipartimenti di Medicina e Chirurgia Traslazionale, Università Cattolica del Sacro Cuore, Rome, Italy; ^5^ CEMAD (Centro Malattie Apparato Digerente)—IBD UNIT—Fondazione Policlinico A. Gemelli IRCCS, Rome, Italy; ^6^ CNIS Research Center for Nanotechnology Applied to Engineering, Sapienza University of Rome, Rome, Italy

**Keywords:** microbiota, cirrhosis, inflammatory bowel disease, nonalcoholic fatty liver disease, liver cancer, antibiotics, exercise, diet

**GRAPHICAL ABSTRACT:**
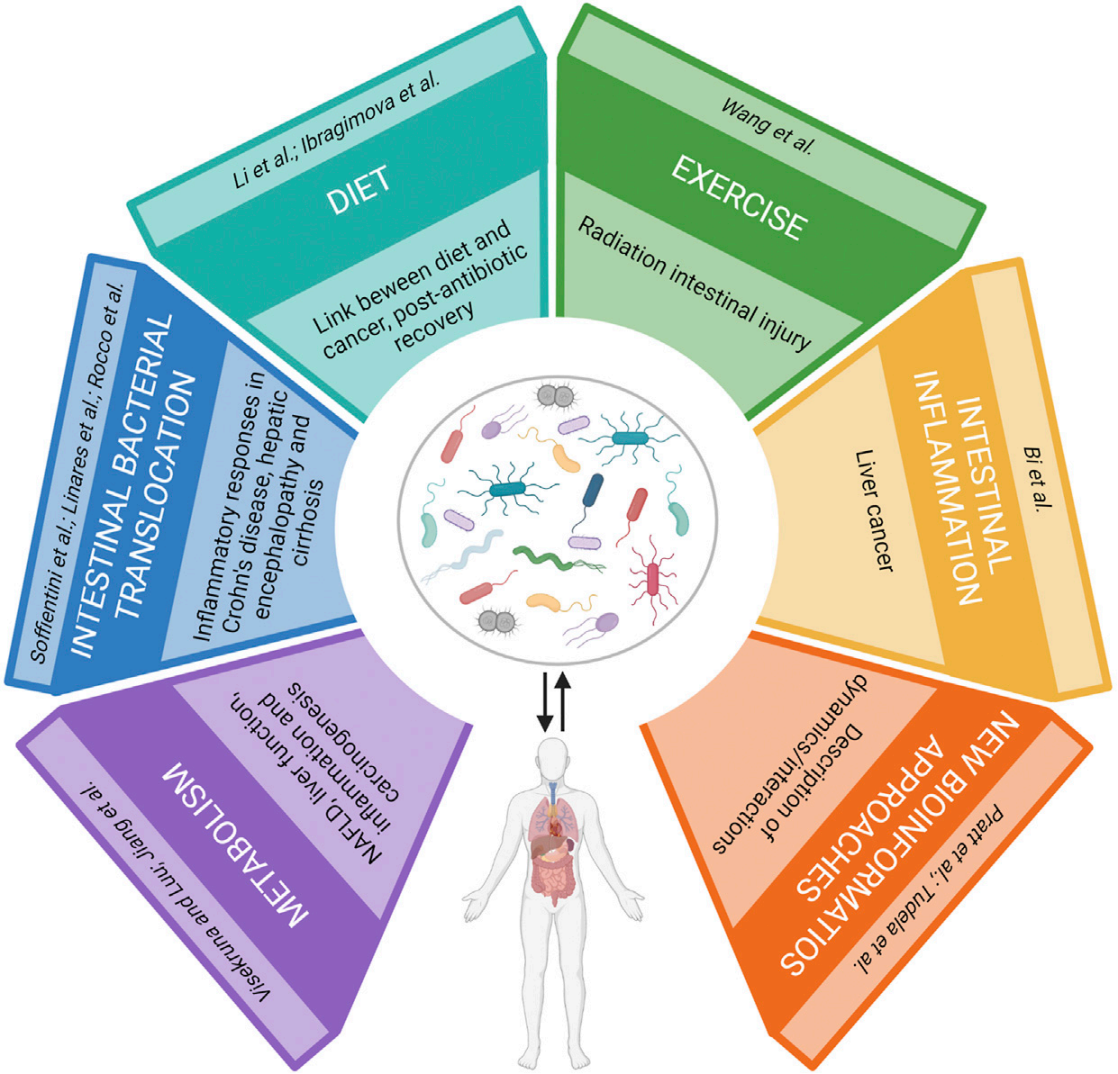


In the last decades, numerous studies highlighted the huge taxonomic and functional complexity that characterizes the human microbiota, demonstrating its role in several physiological processes necessary for host survival. This evidence supports the hypothesis that the microbiota constitutes an “essential organ” instead of a “simple” conglomerate of microbial symbionts. Furthermore, findings point out a mutual host-microbiota interaction, whose imbalance can trigger dysbiosis and, in turn, the onset of illnesses or vice versa. In the latter case, dysbiosis can magnify sicknesses. Several factors can alter microbiota homeostasis and the present Research Topic collects seven reviews and four original contributions focused on the cellular and molecular mechanisms involved in the interaction between host and microbiota which can help to unravel the possible cause of disease and find new therapeutic approaches.

Soffientini et al. and Bi et al. describe new cellular and molecular mechanisms that strengthen the role of the gut microbiota in pathogenesis and progression of liver diseases.

Using a mouse model, Soffientini et al. reported that the deficiency of caspase-11, a protease involved in the intracellular LPS sensing and triggering cell death pathways, gives protection against multi-organ injury induced by low-dose injection of LPS in CCl4-induced hepatic fibrosis. Furthermore, they found that high levels of the human orthologue, caspase-4, in the liver of patients with acute decompensation of cirrhosis is correlated with the degree of injury and clinical outcome. Overall, these data showed for the first time a causal relationship between translocation of gut-derived bacterial products and multi-organ injury in cirrhosis.

Bi et al. provided theoretical support for future clinical practice, discussing the more recent findings regarding the immuno-molecular mechanisms of the gut microbiota and their metabolites in the occurrence and development of liver cancer. They pointed out that a balanced composition in the gut microbiota is able to improve chemotherapy treatment in liver cancer and to reduce adverse reactions.

Furthermore, in this Research Topic recent findings are reported on the identification of either new molecules derived from both bacteria (Visekruna and Luu) and the diet (Ibragimova et al.; Li et al.) or host-microorganism cellular interaction (Jiang et al.; Linares et al.; Rocco et al.) that act as modulators on the host-microbiota interplay and are involved in the development of cancer, intestine and liver diseases. Also, Wang et al. provides new clues regarding the recovery of microbiota after antibiotic therapy and the role of low-intensity exercise in its balance.

Visekruna and Luu discussed the molecular and cellular mechanisms by which short-chain fatty acids and bile acids as dominant classes of bacterial metabolites impact intestinal and liver function, inflammation, and carcinogenesis. The authors examined numerous mechanisms, including epigenetics and the more classical ligand-receptor ones, highlighting current gaps in the field and providing input on possible therapeutic interventions.

Li et al. questioned whether diet can affect the post-antibiotic recovery of the gut microbiome and host metabolism. Indeed, excessive antibiotics exposure leads to various detrimental impacts on host metabolism resulting from an imbalanced gut microbiome. In a mouse model, an integrated metagenomic and transcriptomic approach was used to demonstrate that the effects of an antibiotic intervention on host metabolism are long-lasting, antibiotic-specific, and diet-dependent. Furthermore, it was found that a high-fat diet could worsen the host metabolism recovery from short-term antibiotic perturbation in an antibiotic specific fashion, thus emphasizing the crucial role played by nutrition during post-antibiotic recovery.

Ibragimova et al. reviewed the emerging concepts regarding the relationship between diet, the microbiome, and cancer. They discuss the growing evidence indicating that a primary link between diet and cancer is mediated through dietary constituents influencing the composition and function of the gut microbiome. Furthermore, they underscore that future cancer prevention and treatment should possibly focus on optimizing favourable gut microbiomes and metabolomes.

Jiang et al. investigated the role of prolyl endopeptidase (PREP), an enzyme involved in the gut metabolic homeostasis, finding new clues regarding the crosstalk between gut microbiota and pathogenesis and progression of non-alcoholic fatty liver disease (NAFLD). In a mouse model of NAFLD induced by a high-fat diet, they described that the PREP-gene disruption can promote the abundance of several beneficial butyrate-producing bacteria and reduce hepatic inflammation, ameliorate liver lipogenesis and AMPK/SIRT1 signalling (involved in hepatic steatosis). They proposed PREP as a possible target for NAFLD.

Linares et al. reviewed the current literature regarding the interaction between intestinal bacterial translocation and the development of inflammatory responses in Crohn’s disease. They outlined several factors that contribute to an uncontrolled bacterial translocation in patients with Crohn’s disease, such as dysbiosis, increased permeability of the intestinal barrier, altered immune response, and a genetic predisposition. They also focused in their discussion on the loose response to anti-TNF-alpha biologic therapy observed in patients with Crohn’s disease and the role of bacterial translocation as a contributing factor.

Rocco et al. focused on the role of the gut milieu in the pathogenesis of hepatic encephalopathy. They critically reviewed the latest research findings in the field highlighting novelty and limitations and discussed the therapeutic options and novel treatment strategies.

Wang et al. studied the possible protective role of low-intensity exercise against radiation enteritis. They showed that in a mouse model walking and other comfortable forms of exercise can mitigate the radiation-induced gastrointestinal tract toxicity by restructuring the gut microbiota configuration. More specifically, they found that walking elevates the frequency of *Akkermansia muciniphila* in the digestive tract after radiation exposure and, furthermore, oral gavage of *Akkermansia muciniphila* protects against intestinal radiation toxicity. Their results suggest that *A. muciniphila* can be a useful agent to mitigate the radiation intestinal injury of patients who are clinically unfit to exercise.

In addition to wet lab experiments, bioinformatic based approaches have much to offer to microbiota research. Here, it is also highlighted the need to develop new bioinformatics approaches that describe the dynamics/interactions between the microbial population rather than the mere composition of the microbiota (Pratt et al.; Tudela et al.).

Pratt et al. illustrated the current “-omics” technologies for the study of the gut microbiome in order to identify metabolic biomarkers and patterns. Moreover, based on the results derived by other “-omics” studies, they discussed the significance of biological markers in the homeostasis and immune signalling pathways that affect inflammation or tumour development in the gut microenvironment. In particular, they focused on short-chain fatty acid and bile acid metabolism, inflammasome activation, and cytokine regulation in the context of inflammatory bowel disease and colorectal cancer.

Tudela et al. proposed that there is a need to identify specific keystone members of the gut microbiota ecosystem that carry essential functions that support a healthy symbiotic interaction with the host. Indeed, they underscored the weakness of current bioinformatic tools that focus on “presence or absence” information and do not provide a view of species interactions, thus missing the microbiota dynamics in disease status.

In this Research Topic, we discuss the latest findings and our current understanding of molecular and cellular mechanisms involved in the host-microbiota interaction and disease onset, improving the state of the art and emphasizing the need for further study.

